# Intraspecific genetic variation in host vigour, viral load and disease tolerance during Drosophila C virus infection

**DOI:** 10.1098/rsob.230025

**Published:** 2023-03-01

**Authors:** Megan A. M. Kutzer, Vanika Gupta, Kyriaki Neophytou, Vincent Doublet, Katy M. Monteith, Pedro F. Vale

**Affiliations:** ^1^ Institute of Evolutionary Biology, School of Biological Sciences, University of Edinburgh, UK; ^2^ Institute of Immunology and Infection Research, School of Biological Sciences, University of Edinburgh, UK

**Keywords:** tolerance, resistance, Drosophila C virus, viral infection

## Abstract

Genetic variation for resistance and disease tolerance has been described in a range of species. In *Drosophila melanogaster*, genetic variation in mortality following systemic Drosophila C virus (DCV) infection is driven by large-effect polymorphisms in the restriction factor *pastrel (pst)*. However, it is unclear if *pst* contributes to disease tolerance. We investigated systemic DCV challenges spanning nine orders of magnitude, in males and females of 10 *Drosophila* Genetic Reference Panel lines carrying either a susceptible (S) or resistant (R) *pst* allele. We find among-line variation in fly survival, viral load and disease tolerance measured both as the ability to maintain survival (mortality tolerance) and reproduction (fecundity tolerance). We further uncover novel effects of *pst* on host vigour, as flies carrying the R allele exhibited higher survival and fecundity even in the absence of infection. Finally, we found significant genetic variation in the expression of the JAK-STAT ligand *upd3* and the epigenetic regulator of JAK-STAT *G9a.* However, while *G9a* has been previously shown to mediate tolerance of DCV infection, we found no correlation between the expression of either *upd3* or *G9a* on fly tolerance or resistance. Our work highlights the importance of both resistance and tolerance in viral defence.

## Graphical abstract

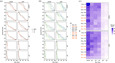

## 1. Introduction

Why do some hosts succumb to infection while others survive? Host heterogeneity in infection outcomes can be attributed in part to two distinct but complementary mechanisms, which together act to maintain host health: mechanisms that limit pathogen growth and mechanisms that prevent, reduce or repair the tissue damage caused during infection without directly affecting pathogen load. The relative balance between these mechanisms may result in phenotypically distinct outcomes. We tend to associate a strong capacity to clear infection with a ‘resistance’ phenotype, while hosts with efficient damage limitation mechanisms may appear to be relatively healthy even if their ability to clear is not pronounced and pathogen loads remain high—generally described as a ‘disease tolerance’ phenotype [1–7].

Beyond differences in their underlying mechanisms, resistance and tolerance can have profoundly different epidemiological and evolutionary outcomes [8–13]. If disease tolerance improves host survival, the infectious period is prolonged, thus increasing pathogen transmission and infection prevalence. In this case, hosts with an allele that confers mortality tolerance (high survival relative to their pathogen load) have a fitness advantage, so the tolerance allele spreads throughout the host population, leading to the eventual fixation of tolerance in the population [14]. However, this prediction contrasts with many studies that find evidence for genetic variation in disease tolerance within a population [15–19]. One possible explanation for this divergence between predicted and observed levels of genetic variation is that disease tolerance may incur fitness costs that are not captured in models of tolerance evolution [14,20]. Further, if disease tolerance acts only to maintain or improve host fecundity, it should be neutral with respect to pathogen prevalence because host lifespan is unaffected, thus the pathogen's transmission period is neither prolonged nor shortened [14]. Therefore, theoretical predictions suggest that we might expect to observe heterogeneity for fecundity tolerance but not mortality tolerance in natural populations [14].

Here, we tested how two intrinsic sources of variation—genetic background and sex—interact to contribute to host heterogeneity in disease defence measured as resistance and tolerance. We focused on the interaction between the fruit fly *Drosophila melanogaster* and Drosophila C virus (DCV), a horizontally transmitted, positive sense RNA virus, that naturally infects multiple *Drosophila* species [21–24]. Systemic infection with high doses of DCV leads to infection of the smooth muscles around the crop, which causes pathology and results in intestinal obstruction, reduced metabolic rate and reduced locomotor activity [25–28]. The majority of genetic variance in host mortality during DCV infection is controlled by large-effect polymorphisms in and around the *pastrel* (*pst*) gene, a viral restriction factor [29–31]. The protective effect of *pst* was confirmed by loss-of-function mutants and an overexpression study [29,32]. However, it is unclear if variation in the protective effects of *pst* acts only by increasing the fly's ability to clear the viral infection, or also to tolerate its pathological effects. As disease tolerance relates to a reduction of pathology independent of pathogen clearance, tolerance mechanisms described to date have included those that prevent, limit or repair tissue damage [1,33–37]. Inflammation is one common cause of such damage during infection. Pro-inflammatory cytokines tend to be associated with decreased tolerance to infection—for example, a tolerant house finch population (*Haemorhous mexicanus*) infected with a bacterial pathogen, *Mycoplasma gallisepticum,* exhibited lower cytokine expression compared with a less tolerant population [33]; mice receiving the anti-inflammatory drug Ibuprofen showed improved tolerance during *Mycobacterium tuberculosis* infection [38]; and lower levels of circulating pro-inflammatory cytokines are associated with tolerance of malaria after re-exposure to the parasite [39]. Negative regulation of immune responses that minimize inflammation would therefore appear to be prime candidates for mechanisms that promote disease tolerance [37,39–41]. This is supported by previous work showing that the epigenetic modifier, *G9a*, which regulates JAK-STAT signalling to prevent hyperactivation of the immune response, increases tolerance to RNA virus infection by limiting immunopathology [42,43]. Further, DCV infection is associated with increased fecundity as well as accelerated developmental time in larvae at both lethal and sublethal doses [27]. Since *D. melanogaster* may tolerate infections by increasing their reproductive output and/ or improving survival outcomes, we used lines that varied in their susceptibility to DCV infection [44] in order to capture the entire range of genetic variation in resistance and tolerance available across the *Drosophila* Genetic Reference Panel (DGRP) panel.

We used males and females flies from 10 DGRP lines [45]: five with a resistant (R) *pst* allele and five with a susceptible (S) allele. We systemically challenged male and female flies with five doses of DCV. We measured fly lifespan and viral load in both sexes, as well as cumulative fecundity and reproductive rate in females. By doing so, we were able to characterize natural variation in resistance, mortality tolerance and fecundity tolerance to DCV. Tolerance is frequently measured as a reaction norm, where host fitness is regressed against parasite load assayed at a fixed dose [2,5]. Instead of relying on host heterogeneity at a single dose, we regressed host lifespan and cumulative fecundity against five viral doses spanning nine orders of magnitude, to examine variation in mortality and fecundity tolerance (see also [46,47]). This allowed us to assess how each fly genotype and sex contribute to host defence across a broad range of infection intensities. In addition to characterizing variation in resistance to and tolerance of DCV infection, we aimed to link this variation with potential mechanisms, particularly for disease tolerance, where knowledge of the underlying mechanisms has lagged behind the description of their phenotypic effects. We therefore also investigated if variation in resistance or tolerance in the tested lines were associated with the expression of either *G9a* or of *upd3*, a JAK-STAT pathway target gene that encodes a cytokine-like protein [48].

## 2. Materials and methods

### 2.1. *Drosophila melanogaster* culture conditions and experimental lines

To assess genetic variation in resistance and tolerance to DCV, we chose 10 lines from the DGRP [45] spanning the range of variation in fly survival within the DGRP when infected systemically with DCV [30,44]. Because the viral restriction factor, *pst* is known to affect survival to DCV infection, we specifically selected five susceptible (S) lines (RAL-138, RAL-373, RAL-380, RAL-765, RAL-818) and five resistant (R) lines (RAL-59, RAL-75, RAL-379, RAL-502, RAL-738) [44,49,50]. The resistant *pst* allele results from a non-synonymous substitution (A/G; Threonine → Alanine) in the coding region of the gene [29]. All lines were previously cleared of *Wolbachia* infection, as it is known to confer protection against DCV [51–53]. All fly stocks in the laboratory, including the DGRP panel, are routinely checked for several viral pathogens using PCR as described in [54]; here we tested for the presence of the common laboratory contaminants DCV, DAV, Nora virus and sigma virus, and no viral contamination was detected (see electronic supplementary material, table S1 for PCR primers). All lines were maintained on standard cornmeal medium [55] at 25°C on a 12 h : 12h light : dark cycle.

### 2.2. Virus preparation

DCV was grown in a *Drosophila* S2 cell culture as described previously [28,56]. The homogenized culture was passed through a sucrose cushion, ultracentrifuged and re-suspended in 10 mM Tris-HCl (pH 7.3). The suspended virus was stored at −80°C in 10 µl aliquots. Virus loads were measured using quantitative real-time PCR as described previously [42]. Briefly, total RNA was extracted using TRI reagent (Ambion) and then reverse transcribed using M-MLV Reverse Transcriptase (Promega) and random hexamers. The manufacturer's protocol was followed to synthesize cDNA. Ten-fold serial dilutions of this cDNA was done up to 10^−10^ dilution. The number of DCV copies in these samples was quantified using DCV specific primers (DCV_Forward: 5′AATAAATCATAAGCCACTGTGATTGATACAACAGAC 3′, DCV_Reverse: 5′ AATAAATCATAAGAAGCACGATACTTCTTCCAAACC 3′) and Fast SYBR green (Applied Biosystems) based qRT- PCR (Applied Biosystems StepOne Plus). The dilution at which no copies were detected was set as zero reference. The viral quantity was back calculated from this point and viral copies in the stock were estimated to be 10^9^ DCV RNA copies per ml.

### 2.3. Survival and cumulative fecundity

All experimental flies were reared under constant density of between 80 and 100 eggs per vial for at least two generations. We infected 3- to 5-day-old adult male and female flies with five concentrations of DCV inoculum: 10^3^, 10^5^, 10^6^, 10^8^ and 10^9^ DCV RNA copies per ml. All the viral inoculums were obtained by diluting the same viral stock solution in sterile 10 mM Tris-HCl. Flies were infected systemically by intra-thoracic pricking using a needle (Minutein pin, 0.14 mm) dipped in the viral suspension. A control group was pricked with a needle dipped in sterile 10 mM Tris-HCl (pH 7.3). In total, we infected 20 individual replicate flies for each combination of DGRP line, DCV concentration and sex, resulting in a total of 2400 flies (20 replicates × 10 DGRP lines × 6 DCV concentrations × 2 sexes). Given the large number of infections (5 replicates per line × dose × sex, approx. 600 flies per day), we blocked the experiment across four days and collected eggs separately from each of the ten DGRP lines on each day. Each fly was housed individually in a vial after infection and flies were monitored for mortality daily. Flies were transferred to new food vials every week until day 30 post-infection, while the previous vials were stored at 25°C until all progeny eclosed as adults. We quantified the cumulative fecundity of each individual fly as the total number of adult offspring produced during this 30-day period (or until death, if this happened prior to the 30th day).

### 2.4. Viral load

In addition to the 2400 flies exposed to DCV to monitor survival, a further five individuals for a given line × dose × sex combination (600 flies in total) were infected to measure the viral load, measured as DCV copies per fly, at 3 days post-infection (3 DPI). We chose this time-point because we wanted to quantify viral load in the flies before the onset of mortality due to infection across all doses, as flies in the higher DCV concentrations started dying within 4 days of infection. Each fly was transferred to TRI reagent at 3 DPI, and flies were frozen at −80°C until RNA extraction. We measured viral load as described previously in [42]. Quantification of viral load by qRT-PCR is often quantified as the ‘genome equivalent’, because here, RT-PCR measures RNA genome copies of at least a partial viral genome (i.e. the equivalent). We generated the DCV standard curve by quantifying DCV load in serially diluted samples of DCV. This standard curve was used for absolute quantification of virus load in the fly samples.

### 2.5. Gene expression

The JAK-STAT pathway has been described previously as being involved in the response to DCV [43]. To test if measures of resistance or tolerance were correlated with the expression of JAK-STAT pathway genes, we pricked 3- to 7-day-old flies with 10 mM Tris-HCl (pH 7.3) (control) or 10^7^ DCV RNA copies per ml. We used 10^7^ DCV RNA copies per ml because it reflected the half maximal effective concentration (EC50) across the 10 tested lines and elicits an immune response in *D. melanogaster* at this dose. Following infection, the flies were housed by line × treatment × sex in vials containing standard Lewis Cornmeal medium. Three days post-infection, we set up five replicates of each treatment combination containing three live flies in 1.5 ml Eppendorf tubes. We anaesthetized the flies on ice, placed them in 60 µl of TRIzol reagent (Invitrogen) and stored them at −70°C for gene expression analyses.

To quantify the differences in transcription levels of *G9a* and the JAK-STAT pathway gene *upd3*, we used quantitative Reverse Transcription PCR (RT-qPCR). First, we homogenized flies submerged in TRIzol Reagent using a pestle motor. Total RNA was extracted using a Direct-zol RNA Miniprep kit (Zymo Research) in accordance with the manufacturer's instructions and stored at −70°C. We included a DNase treatment step per the manufacturer's recommendation, to digest genomic DNA. The isolated RNA was reverse transcribed with M-MLV reverse transcriptase (Promega) and random hexamer primers (ligation at 70°C for 5 min, cDNA synthesis at 37°C for 1 h), diluted 1 : 7 with triple-distilled water and stored at −20°C. Gene expression was quantified using Fast SYBR Green Master Mix (Applied Biosystems) and the primers detailed in the electronic supplementary material, table S1, on the Applied Biosystems StepOnePlus instrument using the following protocol: 95°C for 2 min, followed by 40 cycles of denaturation at 95°C for 10 s and annealing and amplification at 60°C for 30 s. We normalized gene expression of the target genes with the reference gene *rp49* and reported expression as fold change relative to the control flies. We calculated fold change in gene expression as 2-ΔΔCt [57].

To correct for the systematic error among qPCR plates (*n* = 10), we used two calibrators (male Ral-501, replicate 1, infected; male Ral-501, replicate 1, uninfected). Eight microlitres of aliquots were stored at −20°C for later use. The calibrators' mean Ct values were used to calculate correction factors per run, per target gene. Between-plate variation was removed prior to calculating relative gene expression, as described by [58]. Missing values for the *G9a* calibrators for one plate were determined from the correlation of *G9a* expression from all runs between calibrators’ mean Ct and Ct values of samples.

### 2.6. Statistical methods

Statistical analyses were performed in R v.4.0.4 and R Studio 1.4.1106. All data and code is available at https://doi.org/10.5281/zenodo.6651851 [59]. Models 1a and 1b were analysed with a Cox mixed effects survival model using the coxme function in the coxme package [60]. We used Gamma glms (glm function in R base stats package) to evaluate Models 2a and 2b and multiple linear regressions (lm function in the R base stats package) to evaluate Models 5a–8b. Generalized linear mixed models (Models 3a, 3b, 4a and 4b) were analysed using the glmmTMB function with negative binomial error structures with a quadratic parameterization (nbinom2) for Models 3a and 3b, or with a linear parameterization (nbinom1) and zero inflation for Models 4a and 4b [61]. Models 4a and 4b included lifespan as an offset term to control for its effects on cumulative fecundity. All analyses started with the full factorial model and we proceeded to model reduction using model selection criteria [62] and using the check_model function in the performance package if applicable. We tested for significant interactions and/or main effects using type 2 or 3 Wald *χ*^2^ or F tests [63] as appropriate. Experimental block was included as a random effect in Models 1a, 1b, 3a, 3b, 4a and 4b. Interactions were excluded from the final models if *p* < 0.1 unless keeping the interaction resulted in a better model fit (e.g. model no. 6b). Models are further described in tables 1–4 and individual model parameter estimates are included in the electronic supplementary material, tables S2–S17 within appendix S1. Correlations were assessed using Kendall's *tau* coefficient (electronic supplementary material, figures S1 and S4–S11). In figure 3*c*, the *y*-intercept of each function was standardized at 0 to account for differences in general vigour (e.g. [5]) before integration.

**Table 1.** The effects of DCV dose, sex and *pst* or DGRP line on lifespan and mortality tolerance. Model 1a tested survival differences between R and S *pst* alleles and Mode lb tested survival differences among DGRP lines Values in italics are statistically significant. Model details are provided in the electronic supplementary material, appendix S1.

response	model no.	predictor	d.f.	*χ*^2^	*p*-value
lifespan	1a	dose	1	948.7459	*<0.0001*
		*pst* allele	1	28.103	*<0.0001*
		sex	1	2.8748	0.0899
	1b	dose	1	110.1135	*<0.0001*
		line	9	44.4518	*<0.0001*
		sex	1	3.2453	0.0716
		dose × line	9	20.9098	*0.0131*
		line × sex	9	29.9929	*0.0004*

**Table 2.** The effects of DCV dose, sex and *pst* or DGRP line on resistance. Values in italics are statistically significant. Model details are provided in the electronic supplementary material, appendix S1.

response	model no.	predictor	d.f.	*F*	*p*-value
log_10_(Titre)	2a	log_10_(dose)	1	417.904	*<0.0001*
		*pst* allele	1	8.897	*0.0007*
		sex	1	0.004	0.953
		log_10_(dose) × *pst* allele	1	6.751	*0.004*
	2b	log_10_(dose)	1	75.571	*<0.0001*
		line	9	6.043	*<0.0001*
		sex	1	0.009	0.925
		log_10_(dose) × Line	9	3.626	*<0.0001*

**Table 3.** The effects of DCV dose and *pst* or DGRP line on mortality tolerance and fecundity tolerance. Values in italics are statistically significant. Models 3a and 3b tested for differences in mortality tolerance between *pst* alleles or among DGRP lines (indicated by a statistically significant interaction with dose or dose^2^). Models 4a and 4b tested for differences in fecundity tolerance between *pst* alleles or among DGRP lines. Model details are provided in the electronic supplementary material, appendix S1.

response	model no.	predictor	d.f.	*χ*^2^	*p*-value
lifespan	3a	log_10_(dose)	1	38.227	*<0.0001*
		log_10_(dose^2^)	1	510.012	*<0.0001*
		*pst* allele	1	38.6731	*<0.0001*
		sex	1	2.7915	0.09477
	3b	log_10_(dose)	1	1.429	0.232
		log_10_(dose^2^)	1	45.451	*<0.0001*
		line	9	55.658	*<0.0001*
		sex	1	0.367	0.545
		log_10_(dose) × line	9	33.009	*0.00013*
		1og_10_(dose^2^) × line	9	36.088	*<0.0001*
		line × sex	9	20.872	*0.013*
cumulative fecundity	4a	log_10_(dose)	1	5.5437	*0.0186*
		*pst* allele	1	97.5767	*<0.0001*
	4b	log_10_(dose)	1	2.1671	0.141
		line	9	88.8993	*<0.0001*
		dose × line	9	16.9953	*0.0488*

**Table 4.** The effects of sex and *pst* or DGRP line on baseline (relative to rp49) and infected *G9a* or *upd3* expression. Values in italics are statistically significant. Model details are provided in the electronic supplementary material, appendix S1.

response	model no.	predictor	d.f.	*F*	*p*-value
baseline *G9a* expression	5a	*pst* allele	1	1.972	0.1634
		sex	1	381.715	*<0.0001*
	5b	line	9	24.087	*<0.0001*
		sex	1	136.325	*<0.0001*
		line × sex	9	1.984	0.0519
infected *G9a* expression	6a	*pst* allele	1	12.2295	*0.0007*
		sex	1	0.6344	0.4277
	6b	line	9	2.2359	*0.0277*
		sex	1	0.1019	0.7504
		line × sex	9	1.4423	0.18439
baseline *upd3* expression	7a	*pst* allele	1	12.448	*0.0006*
		sex	1	49.137	*<0.0001*
	7b	line	9	3.278	*0.0019*
		sex	1	29.133	*<0.0001*
		line × sex	9	2.334	*0.0217*
infected *upd3* expression	8a	*pst* allele	1	0.8945	0.3466
		sex	1	5.1221	*0.0259*
		*pst* allele × sex	1	3.1105	0.081
	8b	line	9	4.2044	*0.0002*
		sex	1	0.0857	0.7704
		line × sex	9	3.1997	*0.00235*

## 3. Results

### 3.1. *pastrel* affects fly survival during infection and vigour in the absence of infection

**Figure 1.**
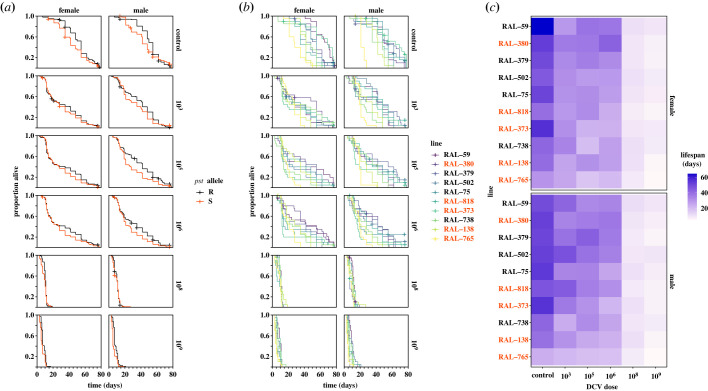
Effects of *pst*, genetic variation, sex and viral dose on survival up to 78 days post-infection. Flies were sham treated (control) or infected with one of five doses (10^3^, 10^5^, 10^6^, 10^8^, 10^9^) of Drosophila C Virus. (*a*) Survival in resistant (R) and susceptible (S) line types. (R lines: RAL-59, RAL-75, RAL-379, RAL-502, RAL-738; S lines: RAL-138, RAL-373, RAL-380, RAL-765, RAL-818). Uninfected resistant lines have a survival advantage in comparison to susceptible lines. Survival tends to improve later in life at low to intermediate infection intensities, but this effect is nearly absent at high DCV doses. (*b*) Each Kaplan–Meier curve represents the cumulative survival of 20 individuals. Viral dose is logged for ease of interpretation. (*c*) Heatmaps showing mean lifespan for female (i) and male (ii) flies, where DGRP lines are arranged according to mean total survival time of males and females. There were differential effects of both line and dose and line and sex on survival after viral infection (*b*) and (*c*). R lines are shown in black and S lines are shown in dark orange. For statistics, see table 1.

First, we examined the effects of DCV dose and sex on survival across 10 genotypes to determine if hosts varied in their susceptibility to viral infection. Because *pst* is known to affect fly mortality following DCV infection, we selected five S lines (138, 373, 380, 765, 818) and five R lines (59, 75, 379, 502, 738), based on previously described infected lifespans [30]. As expected, R lines tended to live longer than S lines (figure 1*a*, table 1, Model 1a, *pst* allele: *p* < 0.0001), though this was also the case in the absence of infection (i.e. general vigour). Examining all 10 lines separately, we detected genetic variation in survival and found that the 10 tested lines differed in their responses to dose (figure 1*b,c*; table 1, Model 1b, dose × line: *p* = 0.013), while sex and genetic background affected survival independently of dose (figure 1*b,c*; table 1, Model 1b, line × sex: *p* = 0.0004).

### 3.2. *pastrel* is associated with variation in the ability to control viral load

**Figure 2.**
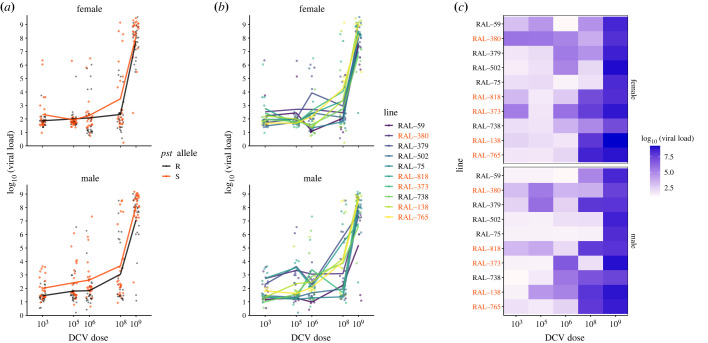
*Drosophila melanogaster* resistance to DCV. (*a*) DCV viral load (DCV copies per fly) in R and S DGRP lines, measured 3 days post-infection (3 DPI). R lines: RAL-59, RAL-75, RAL-379, RAL-502, RAL-738; S lines: RAL-138, RAL-373, RAL-380, RAL-765, RAL-818. DCV load is generally lower in resistant DGRP lines. (*b*) DCV load measured at 3 DPI differs as a function of sex and line and increases as dose increases. Each data point (*n* = 5, line × sex × dose) represents the viral load from a single fly. Values are plotted on log_10_ transformed *x*- and *y*-axes. (*c*) Variation in mean viral load for each level of line and dose. Viral load is logged for clarity. R lines are shown in black and S lines are shown in grey. For statistics, see table 2.

While *pst* has been previously associated with variation in survival following systemic DCV infection, it is not known if *pst* acts only by improving viral clearance, or if flies carrying the resistant (R) alleles are instead better able to tolerate high viral titres. To test this, we quantified resistance as the rate at which viral load increased with increasing doses of viral inoculum. This allows a more complete measure of pathogen burden for each fly line and sex across several orders of magnitude of viral load. Overall, male and female flies with a resistant (R) *pst* allele had significantly lower viral loads compared to susceptible (S) lines (figure 2*a*, table 2, Model 2b, *pst* allele: *p* = 0.0007), indicating that *pst* explains at least some of the variation in viral loads. For all lines and in both sexes, exposure to higher concentrations of DCV resulted in higher viral loads measured 3 days post-infection. However, the magnitude of this increase across DCV doses varied among lines (figure 2*b,c*, table 2, Model 2B, dose × line: *p* < 0.0001).

### 3.3. Mortality tolerance to Drosophila C virus is genetically variable

**Figure 3.**
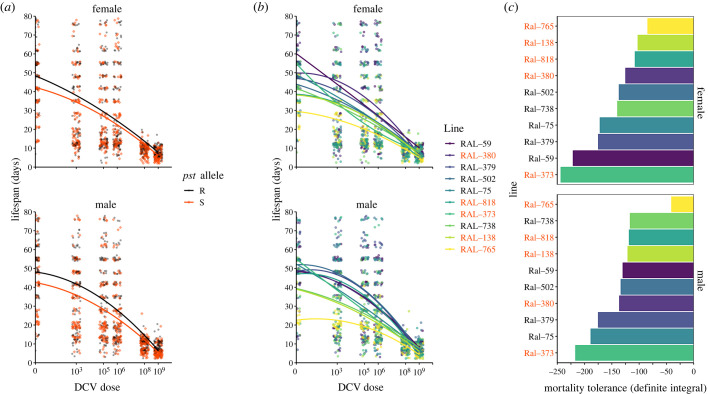
Mortality tolerance in DCV-infected flies shows evidence of genetic variation and nonlinearity. (*a*) Lifespan in resistant (R) and susceptible (S) DGRP lines. Resistant lines tend to live longer than susceptible lines and are equally tolerant to DCV infection. R lines: Ral-59, Ral-75, Ral-379, Ral-502, Ral-738; S lines: Ral-138, Ral-373, Ral-380, Ral-765, Ral-818. (*b*) Reaction norms are plotted for each line and split by sex. We use dose in place of titre (i.e. [42,46]) to estimate variation in tolerance. (*c*) Integrals for each DGRP line, split by sex. The y-intercept of each function was standardized at 0 to account for differences in general vigour (e.g. [5]) before integration. Bars are ordered from least tolerant (Ral-373) to most tolerant (Ral-765).

Since we established that dose was a good indicator of viral load (figure 2), we used dose as a covariate and a proxy for viral load in our tolerance models. First, we examined the effect of *pst* on mortality tolerance and found that flies carrying the R allele tended to maintain higher survival over the range of tested doses (higher intercept in figure 3*a*; table 3, Model 3a; *pst* allele: *p* < 0.0001) but we did not detect an effect of *pst* on mortality tolerance (similar definite integrals, when accounting for differences in the intercept). When analysing how survival changes with increasing concentrations of viral challenge, we observed that there was a quadratic relationship between genotype and dose and found that mortality tolerance to DCV was genetically variable (figure 3*b,c*; table 3, Model 3b; Dose^2^ × Line: *p* < 0.0001). In order to examine differences in tolerance among lines, the y-intercept of each function was standardized at 0 to account for differences in general vigour (e.g. [5]) before integration. Here, a small negative integral value (e.g. Ral-765) indicates a small change in mortality across the tested doses (high tolerance), whereas a large negative integral value (e.g. Ral-373) indicates large changes in mortality across several orders of magnitude of viral exposure (lower tolerance) (figure 3*c*).

### 3.4. Fecundity tolerance during Drosophila C virus infection varies according to fly genetic background

**Figure 4.**
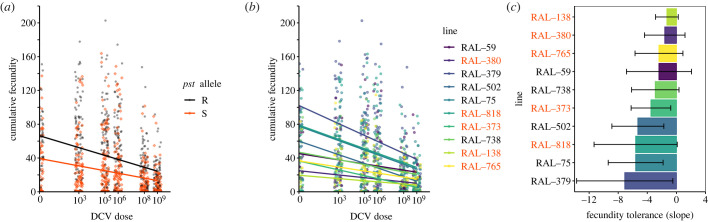
DCV-infected DGRP lines show evidence of genetic variation in fecundity tolerance. (*a*) Cumulative fecundity in R and S DGRP lines. Susceptible lines have fewer offspring than resistant lines regardless of infection status but are equally as tolerant as R lines (similar slopes). R lines: Ral-59, Ral-75, Ral-379, Ral-502, Ral-738; S lines: Ral-138, Ral-373, Ral-380, Ral-765, Ral-818. (*b*) Reaction norms are plotted for each DGRP line. Each data point represents the cumulative fecundity of a single fly during its lifetime. (*c*) Slopes ± s.e. of reaction norms plotted in (*b*). Bars represent the fecundity tolerance of each DGRP line. Lines are ordered from the least tolerant (Ral-379) to most tolerant (Ral-138). For statistics, see table 3.

Hosts may tolerate an infection by limiting its negative effects not only on survival but also on reproduction (known as fecundity or sterility tolerance) [6,14,16,64–66]. We therefore asked if females from the 10 DGRP lines showed variation in fecundity tolerance to DCV. We measured cumulative fecundity (adult offspring production) in single flies over a 30-day period and then quantified fecundity tolerance as the ability to maintain reproduction for increasing viral doses. When accounting for differences in infected lifespan, females with the resistant (R) *pst* allele tended to have more offspring than females with the susceptible (S) allele, (figure 4*a*, table 3; Model 4a, *pst* allele: *p* < 0.0001). This effect occurred regardless of infection status and R and S lines were equally tolerant, indicated by the similar slopes. The fecundity data further suggest that the R allele is associated with improved reproductive fitness even in the absence of infection (figure 4*a*, Dose 0).

In contrast with mortality tolerance, which showed a nonlinear reduction in survival with increasing viral challenge, here the relationship between dose and fecundity was linear and we observed significant differences between fly genotypes in the slopes of these linear relationships (figure 4*b*, table 3, Model 4b; dose × line: *p* = 0.0488), although we note that this effect was only marginally significant. To quantify the extent of this decline, we used the slope for each line, where a shallow slope indicates a small change in fecundity across several orders of magnitude of DCV exposure (figure 4*c*, e.g. Ral-138, Ral-380), while steep negative slopes indicate large changes in fecundity with increasing DCV dose, suggesting low fecundity tolerance (figure 4*c*, e.g. Ral-379). We wondered if we could detect a trade-off between fecundity tolerance and mortality tolerance, as might be expected if investing in fecundity comes at a trade-off with investing in immunity and/or lifespan [67,68]. However, we did not find any evidence of a trade-off between mortality tolerance and fecundity tolerance (electronic supplementary material, figure S1). Overall, our data suggest that the ability to resist or tolerate DCV infection is decoupled in *D. melanogaster*.

### 3.5. *pastrel* affects upd3 expression in the absence of infection and G9a expression in infected lines

**Figure 5.**
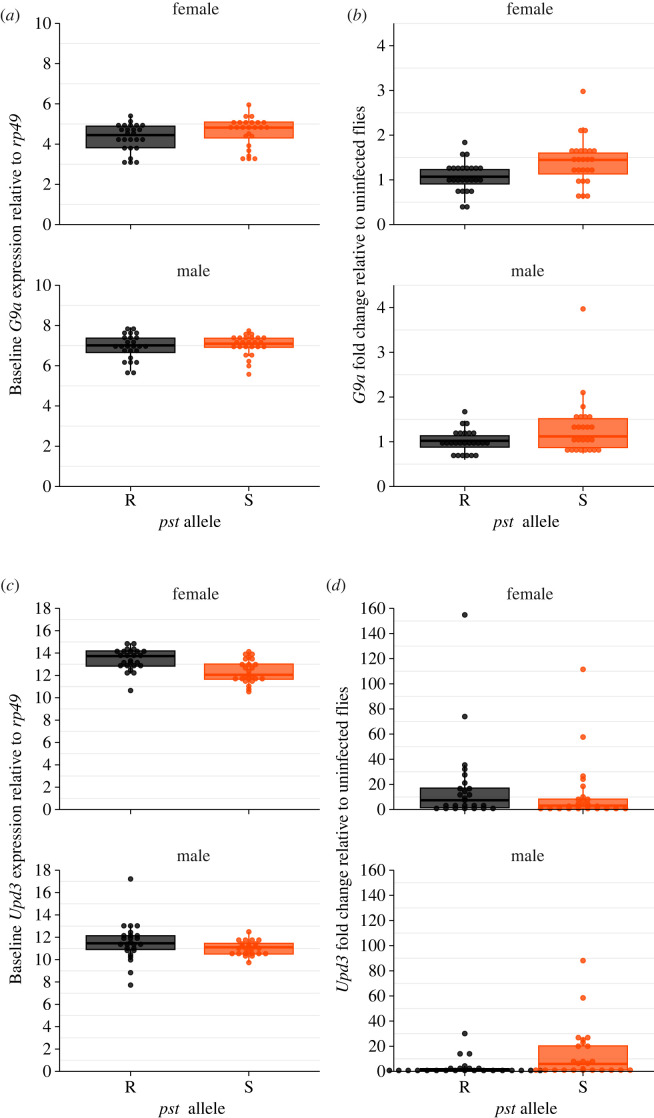
*pst* has differential effects on gene expression between uninfected and infected flies. (*a*) *G9a* expression relative to *rp49* in the absence of infection is not significantly affected by *pst*. (*b*) Infected flies *G9a* expression is higher in (S) susceptible DGRP lines but is unaffected by sex. (*c*) *upd3* expression relative to *rp49* is higher in (R) resistant DGRP lines and tends to be lower in males. (*d*) Sex affects infected expression of *upd3*. R lines: Ral-59, Ral-75, Ral-379, Ral-502, Ral-738; S lines: Ral-138, Ral-373, Ral-380, Ral-765, Ral-818. For statistics, see table 4.

In a separate experiment, we examined *G9a* and *upd3* expression in males and females infected with a viral concentration of 10^7^ DCV IU ml^−1^. We focused on *G9a* because it has been shown to mediate tolerance to DCV infection by regulating the JAK-STAT response [42,43], whereas *upd3* encodes a cytokine-like protein and is the main JAK-STAT ligand induced in response to viral challenge [69]. We reasoned that their expression may explain some variation in disease tolerance and resistance to DCV infection in the 10 DGRP lines (figures 1–3). *pst* status was not associated with differences in baseline *G9a* expression in uninfected flies (figure 5*a*, table 4, Model 5a) but we found that *G9a* expression in infected flies was lower in flies carrying a resistant (R) allele versus those carrying a susceptible (S) allele (figure 5*b*, table 4, Model 6a, *pst* allele: *p* = 0.0007). Baseline *upd3* expression was lower in the S lines (figure 5*c*, table 4, Model 7a, *pst* allele: *p* = 0.0006) but infected flies showed similar levels of *upd3* expression regardless of their *pst* allele (figure 5*d*, table 4, Model 8a).

### 3.6. Genetic variation in the expression of upd3 and G9a does not explain variation in resistance or tolerance

Examining gene expression across all 10 lines, we found evidence of genetic variation in *G9a* expression (electronic supplementary material, figure S2A; table 4; Model 5b, line: *p* < 0.0001), and females tended to have lower baseline expression compared with males (electronic supplementary material figure S2A; table 4; Model 5b, sex: *p* < 0.0001). We found differential effects of sex and line on uninfected *upd3* expression (electronic supplementary material, figure S2B; table 4; Model 7b; line × sex: *p* = 0.022). In infected flies, *G9a* expression varied between fly lines (electronic supplementary material, figure S3A, table 4, Model 6b; line: *p* = 0.028), and males and females differed in their expression of *upd3* following infection, with males showing generally lower *upd3* expression, although the magnitude of these sex differences varied between DGRP lines (electronic supplementary material, figure S3B; table 4; Model 8b; sex × line: *p* = 0.002). While both the baseline and infected gene expression differed among fly lines for *G9a* and *upd3*, we did not detect a significant correlation between the expression of either gene and mortality tolerance or fecundity tolerance (electronic supplementary material, figures S4–S11).

## 4. Discussion

We found evidence of genetic variation in disease tolerance in *D. melanogaster* during systemic infection with DCV, measured both as the ability to maintain survival and reproduction, across a wide range of concentrations of viral challenge. We also confirmed results that the viral restriction factor *pastrel* increases fly survival by reducing viral loads, and we further uncovered previously undescribed effects of *pst* allele status on general fly vigour in the absence of infection, and effects on the expression of the JAK-STAT ligand *upd3* and the epigenetic regulator of JAK-STAT, *G9a*.

### 4.1. *pastrel* affects host vigour in the absence of infection

The restriction factor *pst* has been previously shown to explain most of the variance in fly mortality following systemic DCV infection [30]. Our data confirm these effects, and further confirm that *pst*-mediated increase in fly survival is mainly due to its effects on suppressing DCV titres, which is consistent with its proposed role as a viral restriction factor [29]. The resistant *pst* allele results from a non-synonymous substitution (A/G; Threonine → Alanine) in the coding region of the gene [29]. The susceptible allele is ancestral and has been shown to play some part in antiviral defence, as the overexpression of the allele improves survival after DCV infection and knockdown of the allele makes flies more susceptible to infection. The resulting amino acid substitution is therefore an improvement on an already existing antiviral defence [29].

However, our data also suggest that the effects of *pst* extend beyond viral clearance, as the resistant (R) allele *pst* was associated with a general improvement in fly reproduction and lifespan, even in the absence of infection. To our knowledge, this is the first study to demonstrate *pastrel's* effects on general fly vigour. This result is somewhat surprising, because we might expect a mutation that confers antiviral protection to trade-off against other life-history traits [70], or that an allele conferring both a survival and fecundity advantage should become fixed in the population. In previous work, sham- infected control flies that over expressed the S allele tended to live longer than those that over expressed the R allele, suggesting that overexpression of R comes with costs [29]. That study also found natural variation in *pst* gene expression and that its expression is associated with improved survival outcomes after DCV infection, but it is unclear if this is also associated with improved vigour in the absence of infection. Likewise, in a separate study where flies were selected for survival to DCV, *pst* was also identified as being involved in adaptation to DCV, with no apparent detrimental effects on egg viability, reproductive output or developmental time [71,72]. Our study confirms that the R allele does not seem to carry costs, but is associated with fitness benefits in the absence of infection. Taken together, it is therefore puzzling why the R allele has not risen to fixation, and why S alleles are maintained in the population. It seems likely that the R allele may come with hidden costs that are not manifested under *ad libitum* laboratory conditions. For example, dietary manipulation can sometimes uncover the costs associated with immunity [16,65,70].

### 4.2. *pastrel* controls resistance to Drosophila C virus

While previous studies established that ‘susceptibility’ to DCV is controlled by *pst*, those studies did not directly assay viral loads in resistant versus susceptible natural variants but based their classification on survival data from the DGRP or viral load data from knockdown and over expression experiments. These confirmed that the *pst* gene confers resistance—viral loads were higher in knockdown flies versus controls and overexpression of both S and R alleles increased resistance—but, crucially, they do not establish whether *pst* underlies variation in viral load in natural fly populations [29,30]. Given these results, there were two possibilities: (i) the R allele confers resistance by controlling viral loads or (ii) the R allele confers tolerance to DCV by maintaining survival or reducing damage in the face of infection. Our results support the first possibility that the R allele promotes resistance, demonstrated by lower viral loads in DGRP lines carrying the R allele, and that this protective effect was present in males and female flies, across several orders of magnitude of viral challenge.

### 4.3. Genetic variation in mortality tolerance and fecundity tolerance

Fly genetic background affected the ability of flies to tolerate DCV infection, both when tolerating the mortality caused by infection, and by maintaining fecundity at low and intermediate viral challenge doses. Previous theoretical work showed that variation in fecundity tolerance is more likely to occur if it comes at a cost to host lifespan or another life-history trait [14]. Although we did not observe a trade-off with survival or mortality tolerance in our system, it is possible that fecundity tolerance comes at a cost to another trait that we did not measure. Evidence for genetic variation in both mortality and fecundity tolerance phenotypes is widespread throughout the animal kingdom (reviewed in [2,7]), reinforcing the idea that disease tolerance is an important defence strategy in response to a range of pathogens. It is notable, however, that most experimental studies examining genetic variation in disease tolerance have rarely measured it in the context of viral infections [73]. Our work is, to our knowledge, the first to describe genetic variation in both mortality and fecundity tolerance of a viral infection.

### 4.4. Linear and nonlinear changes in health

The majority of tolerance experiments often assume a linear relationship between pathogen load and host health (or other fitness trait), but there is no reason to assume that health should decrease at a constant rate in relation to pathogen burden [5,42,74–76]. We show that some genotypes maintain their health (measured as lifespan) at low and intermediate DCV doses, whereas health declines rapidly at higher challenge doses. Similar nonlinear relationships between pathogen load and health occur over the course of natural HIV infection in humans [75], in blue tits (*Cyanistes caerulus*) infected with the blood parasite, *Haemoproteus majoris* [76]*,* and in *Drosophila melanogaster* infected with *Listeria monocytogenes* [74] or DCV [42]. By contrast, we found that the relationship between cumulative fecundity and viral dose was best explained by a linear relationship, although previous studies on DCV's effects on fecundity note that offspring production tends to increase at low or intermediate viral doses [27].

### 4.5. No sex differences in tolerance or resistance to Drosophila C virus

Sexual dimorphism in immunity is widespread across metazoans, and to a large extent has frequently been overlooked in experimental studies of infection [77–79]. The sexes can differ in optimal immune investment and allocate resources to different areas of the immune response [8,19,80,81]. In general, females tend to be more immunocompetent than males because they improve their fitness by increasing investment in immune defence [80–82]. In systems where resistance and tolerance are negatively correlated as shown in malaria-infected mice [17], one sex may invest more into resistance, while the other may invest in tolerance. Sex differences in disease tolerance are also predicted to have qualitatively different consequences for pathogen evolutionary trajectories [8].

It was therefore an explicit aim of the present study to quantify sex differences in lifespan, resistance and disease tolerance following DCV infection, to examine potential sexual dimorphism in disease tolerance. However, we were surprised to find that fly sex contributed little to the variation in the disease phenotypes we investigated, particularly viral loads or mortality tolerance. This contrasts with some results from disease tolerance in other host–pathogen systems where sexual dimorphism in tolerance has been observed (reviewed in [8]). For example, males infected with *P. aeruginosa* were more tolerant and resistant than females, with evidence of sexual antagonism for tolerance, indicated by a negative genetic intersexual correlation [19]. By contrast, Gupta *et al.* [27] noted that *D. melanogaster* males are more susceptible than females to systemic DCV infection, while no difference between males and females was detected in tolerance of HIV [75]. It is therefore difficult to make generalizations concerning disease outcomes between the sexes, which will depend on the specific host and pathogen species, particularly as the expression of many infection-related traits is often the outcome of complex interactions between host sex, genetic background and mating status [44]. What is clearer is that work reporting sex-specific infection outcomes are less common than is desirable [77], especially regarding disease tolerance phenotypes.

### 4.6. *pastrel* is associated with changes in pre- and post-infection gene expression

Given previous work [42,43], we expected that *G9a* and *upd3* expression would correlate with disease tolerance and explain some of the phenotypic variation we see among DGRP lines. Although we observed differential effects of genetic background and sex in gene expression, this appeared to be independent of disease tolerance phenotypes. We note that *pst* was associated with differences in baseline *upd3* expression as well as infected *G9a* expression. Baseline *upd3* expression was lower in susceptible lines, suggesting that expression levels prior to infection may dictate the speed or strength of the antiviral immune response. Differences in baseline gene expression have been shown to affect chronic disease outcomes (e.g. rheumatoid arthritis, multiple sclerosis, lung cancer, autoimmune diseases) [78,83–85], so we suggest that basal expression levels may be important predictors of resistance and tolerance. Similarly, infected *G9a* expression was higher in susceptible lines, which may point to differences in the damage control response, but did not detect this as a tolerance phenotype in our experiments. In fact, it is possible that *G9a* expression may not be specifically related to DCV infection, as recent work has highlighted the likely role of this methyltransferase as a master regulator of metabolic homeostasis and tolerance to a variety of biotic and abiotic stressors in many different species [86].

## 5. Concluding remarks

In summary, we describe genetic variation in disease tolerance in *Drosophila* following systemic DCV infection, in males and females, across a range of infectious challenges spanning several orders of magnitude. Further, we find that the *pst* gene is associated with general vigour in the absence of infection and confirm its role in reducing DCV titres during infection. This work offers, to our knowledge, one of the first descriptions of genetic variation in mortality tolerance and fecundity tolerance in a viral infection of invertebrates, adding to the growing effort to describe the causes of host heterogeneity in health and its consequences for pathogen spread and evolution [50,87,88].

## Data Availability

All data and code for analysis can be accessed at: https://zenodo.org/record/6651851 [59]. The data are provided in the electronic supplementary material [89].
